# Normalized EMG Amplitude During Repeated Sit-to-Stand Transfers in Patients with Diabetic Peripheral Neuropathy

**DOI:** 10.3390/bioengineering13070836

**Published:** 2026-07-21

**Authors:** Safi Ullah, Kamran Iqbal, Muhammad Rizwan

**Affiliations:** School of Engineering and Engineering Technology, University of Arkansas at Little Rock, Little Rock, AR 72204, USA; safi@cytoastra.com (S.U.); mrizwan@ualr.edu (M.R.)

**Keywords:** diabetic peripheral neuropathy, sit-to-stand, surface electromyography, neuromuscular activation, transitional movement, functional mobility

## Abstract

Diabetic peripheral neuropathy (DPN) causes progressive sensorimotor dysfunction of the lower extremities, impairing mobility and increasing fall risk. Phase-specific neuromuscular activation during repeated sit-to-stand (STS) transfers in DPN remains poorly characterized. This study compared lower-limb muscle activation during repeated STS transfers in fifteen ambulatory DPN patients and fifteen age- and gender-matched healthy controls. Bilateral surface electromyography (sEMG) was recorded from the vastus lateralis, vastus medialis, biceps femoris, gluteus maximus, and gluteus medius; normalized to maximum voluntary contractions (MVCs), and analyzed separately for STS and stand-to-sit phases. No significant between-group differences were observed for any muscle during either phase (all *p* > 0.05). Effect sizes were small-to-medium, with confidence intervals indicating uncertainty around the estimated between-group differences. Notably, gluteus maximus activation during STS demonstrated the largest between-group effect size, favoring the DPN group (d = 0.54), although this difference was not statistically significant. This finding may indicate a possible proximal hip-extensor strategy that requires confirmation in larger studies. Both groups demonstrated significantly greater vastus lateralis and gluteus maximus activation during STS than stand-to-sit, reflecting greater neuromuscular demand during rising. Overall, the non-significant between-group findings, together with the effect-size and confidence-interval analysis, suggest that MVC-normalized EMG amplitude alone may have limited sensitivity for detecting neuropathic neuromuscular alterations during transitional tasks in ambulatory patients with DPN.

## 1. Introduction

Diabetic peripheral neuropathy (DPN) is one of the most prevalent and disabling complications of diabetes mellitus, affecting up to 50% of patients depending on age and disease duration [1,2]. It is characterized by progressive axonal degeneration and segmental demyelination of peripheral sensory and motor nerves [1,2], resulting in impaired proprioception, reduced lower-limb sensation, muscle weakness, balance deficits, gait impairment, and increased fall risk [1,2,3,4,5]. The World Health Organization reported that approximately 14% of adults aged 18 years and older were living with diabetes in 2022, underscoring the scale of this public health burden [6]. With global diabetes prevalence continuing to rise, the functional consequences of DPN represent an increasingly important target for clinical assessment and rehabilitation.

The neuromuscular consequences of DPN extend beyond sensory dysfunction. Motor axon loss in DPN follows a length-dependent progression, with reduced motor unit numbers documented predominantly in distal lower-limb muscles, including the intrinsic foot muscles and ankle dorsiflexors/plantar flexors, while functional mobility tasks may also involve compensatory demand on more proximal muscle groups, including the quadriceps, hamstrings, and hip abductors/extensors. Critically, however, motor unit loss in DPN typically occurs later in the disease process, following the onset of sensory deficits [7]. In ambulatory patients with preserved functional mobility, gross motor output amplitude may therefore remain relatively intact despite ongoing sensorimotor degradation, as surviving motor units undergo compensatory reinnervation and upregulate their discharge rates. This mechanistic distinction is important when interpreting neuromuscular findings in ambulatory DPN cohorts, as it suggests that normalized electromyographic amplitude, a measure of gross motor output, may have limited sensitivity for detecting early-to-moderate neuropathic impairment. Recent studies using high-density sEMG and quantitative muscle imaging have also identified altered tibialis anterior recruitment and distal lower-limb muscle morphological and mechanical changes in individuals with DPN [8,9].

Previous biomechanical investigations in DPN have primarily focused on gait abnormalities, plantar pressure distribution, balance impairment, and postural control during walking tasks [1,2,10,11]. Patients with DPN commonly demonstrate slower gait velocity, shorter step length, increased gait variability, and impaired balance compared with healthy individuals [1,2]. Surface electromyography (sEMG) studies have further identified altered neuromuscular recruitment patterns during locomotion in DPN, including abnormal muscle activation timing, increased co-activation, and prolonged muscle activity, which contribute to gait instability and fall risk [10,12]. The more recent literature has identified sEMG as a valuable tool for detecting subtle neuromuscular alterations in DPN, including intermuscular coordination changes that may not be apparent through gross functional observation alone [11]. In our previous work on locomotion involving the same clinical cohort, patients with DPN demonstrated slower gait velocity, reduced mobility, and poorer static balance compared with healthy controls, while dynamic postural stability during walking appeared relatively preserved [13]. A recent controlled study combining force-plate, motion-capture, and sEMG measurements during a functional reach task likewise identified neuromuscular adaptations associated with foot somatosensory loss in DPN [14]. These findings suggested that ambulatory patients with DPN may utilize compensatory movement strategies to maintain functional mobility during steady-state locomotion. However, the neuromuscular demands of repeated sit-to-stand transfers differ substantially from those of steady-state gait and postural-control tasks. Whereas our previous work in this cohort focused on gait velocity, mobility, static balance, and dynamic postural stability, the present study examines phase-specific lower-limb EMG amplitude during repeated STS and stand-to-sit transfers. This provides a distinct analysis of transitional movement, a functional context in which rapid movement initiation, rising against gravity, controlled lowering, and postural stabilization may reveal neuromuscular features not captured during gait-based assessment.

Sit-to-stand (STS) transfer is among the most frequently performed functional tasks of daily living and is biomechanically distinct from walking. Successful STS transfer requires coordinated forward trunk movement, lower-limb force production, dynamic postural control, and stabilization of the center of mass over the base of support, imposing substantial neuromuscular demands on the quadriceps, hamstrings, and hip musculature, particularly during the rising phase [15]. Unlike steady-state walking, STS involves rapid transitional movement between stable postures and may therefore expose neuromuscular control deficits not readily observable during gait. Falls most commonly occur during transitional tasks such as STS and stand-to-sit, making their neuromuscular characterization clinically important for fall risk assessment and rehabilitation planning in DPN [16]. Recent neurophysiological investigations have characterized STS as a multi-phase movement involving complex muscle synergies and coordinated activation patterns; however, the available literature has largely focused on healthy individuals or central neurological disorders rather than peripheral neuropathic conditions [17].

Although STS performance tests, such as the five-times STS test and the 30 s chair stand test, are frequently used in DPN rehabilitation and fall-risk assessment, most studies have focused primarily on task completion time or functional performance outcomes rather than the underlying neuromuscular strategies [18,19]. Other recent systematic reviews likewise indicate that balance, strengthening, gait, and multicomponent exercise programs can improve balance-related outcomes and fear of falling in patients with DPN, although evidence regarding reduction in actual fall incidence remains limited [20,21]. Relatively little attention has been directed toward phase-specific lower-limb muscle activation during repeated STS transfers in patients with DPN. It therefore remains unclear whether ambulatory patients with DPN demonstrate altered neuromuscular recruitment during transitional movements despite preserved task completion capability, which is a distinction of clinical importance because successful task completion may conceal compensatory motor-control strategies or altered muscle recruitment patterns associated with neuropathic impairment.

Therefore, the goal of this study was to examine lower-limb muscle activation patterns during repeated STS transfers in ambulatory patients with DPN compared with age- and gender-matched healthy controls. We specifically aimed to investigate whether maximum voluntary contraction (MVC)-normalized sEMG amplitude is sensitive to neuropathic neuromuscular impairment during this transitional task. Surface EMG signals were collected bilaterally from the vastus lateralis, vastus medialis, biceps femoris, gluteus maximus, and gluteus medius during repeated STS transfers and normalized to maximum voluntary contractions (MVCs). Muscle activation amplitudes were analyzed separately during STS and stand-to-sit phases, and effect sizes were calculated for all comparisons to support the interpretation of findings in the context of sample size.

## 2. Materials and Methods

### 2.1. Participants

Fifteen individuals diagnosed with DPN and fifteen age- and gender-matched healthy controls participated in the study. The DPN cohort was identical to the cohort previously investigated in our earlier gait and postural control study [13]. The same participants contributed data to both studies; however, the present analysis addresses a distinct research question, functional task, and outcome set. The earlier publication examined gait and postural control outcomes, whereas the present study analyzes phase-specific lower-limb sEMG activation during repeated STS transfers. No data were pooled across publications, and all statistical analyses reported here were performed only on the STS-specific EMG outcomes. Participants with DPN were recruited from Little Rock Walk-In Clinic (Little Rock, AR, USA) under the supervision of a board-certified endocrinologist. DPN diagnosis was confirmed clinically based on documented type 2 diabetes mellitus, symptom history consistent with peripheral neuropathy, and clinical examination findings indicating distal sensory impairment. Inclusion criteria for the DPN group were: (1) physician-confirmed diagnosis of DPN; (2) minimum 7-year history of type 2 diabetes mellitus; (3) glycated hemoglobin (HbA1c) levels ≥ 7%; (4) absence of additional neurological or musculoskeletal disorders affecting gait or balance; (5) body mass index (BMI) < 35 kg/m^2^; and (6) ability to perform repeated STS transfers independently without assistive devices. Healthy controls were free from known neurological, musculoskeletal, or balance impairments. Participant demographics are presented in Table 1. The study was designed as an exploratory two-group comparison between participants with diagnosed DPN and healthy controls. A non-neuropathic diabetic comparison group was not included in the original study design, and the available DPN sample size was not sufficient to support severity-based subgroup analysis.

Enrollment was constrained by COVID-19 safety restrictions and IRB-imposed limits on in-person research activity during the data collection period (July–October 2021), yielding a sample of 15 participants per group consistent with literature-reported exploratory investigations in this clinical population. All participants provided written informed consent before participation. The study was approved by the Institutional Review Board (IRB) of the University of Arkansas at Little Rock (UALR) and the University of Arkansas for Medical Sciences (UAMS), and all procedures were conducted in accordance with the Declaration of Helsinki. The study is reported with reference to the Strengthening the Reporting of Observational Studies in Epidemiology (STROBE) guidelines for observational studies.

### 2.2. Experimental Procedures

Participants performed repeated STS transfers from a standard foldable steel chair without armrests. The same chair was used for all participants to standardize testing conditions. Chair height was fixed rather than scaled to participant anthropometrics to maintain a consistent clinical STS testing condition across participants. Knee angle at seat-off was not used as a control variable in the present analysis. Participants were instructed to perform five consecutive STS repetitions at a self-selected pace while remaining barefoot throughout testing. Arms were crossed over the shoulders to minimize upper-extremity contribution and isolate lower-limb neuromuscular demand during the task. Reflective markers were worn, and three-dimensional motion-capture data were collected as part of the broader experimental protocol. However, marker-derived kinematic variables were not analyzed in the present STS EMG amplitude study and were not used to derive the reported EMG outcomes. The present manuscript focuses on surface EMG activation amplitudes recorded during repeated sit-to-stand and stand-to-sit phases.

**Figure 1:**
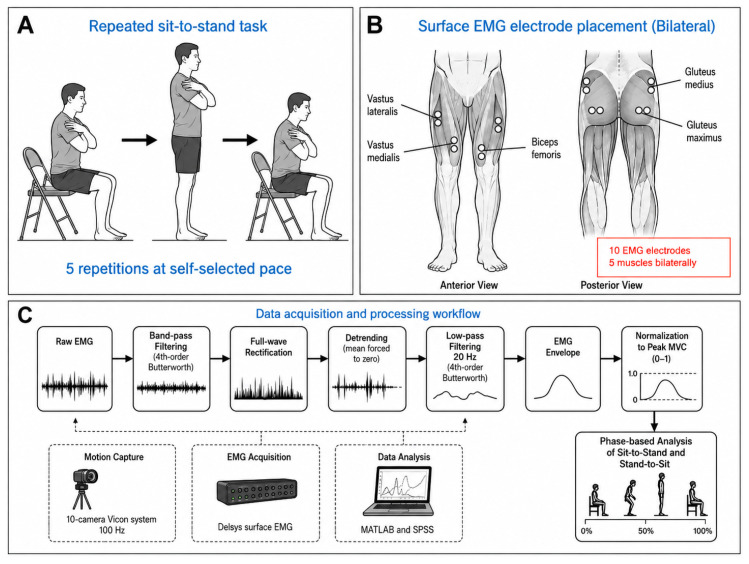
Experimental setup and data-processing workflow for repeated sit-to-stand (STS) transfer analysis. (**A**) Participants performed five repeated STS transfers from a standard chair at a self-selected pace, barefoot and with arms crossed over the shoulders to minimize upper-limb assistance. (**B**) sEMG electrodes were placed bilaterally on five lower-limb muscles: vastus lateralis, vastus medialis, biceps femoris, gluteus maximus, and gluteus medius. (**C**) EMG data-processing workflow. Raw EMG signals were band-pass-filtered using a fourth-order Butterworth filter, full-wave-rectified, detrended, low-pass-filtered at 20 Hz using a fourth-order Butterworth filter to generate EMG envelopes, and normalized to peak MVCs. Normalized EMG amplitudes were then analyzed separately during the STS and stand-to-sit phases. Reflective markers were worn as part of the broader experimental protocol; however, marker-derived kinematic variables were not used in the present EMG amplitude analysis.

Surface EMG electrodes (Delsys Inc., Natick, MA, USA) were used to record muscle activity bilaterally from the vastus lateralis, vastus medialis, biceps femoris, gluteus medius, and gluteus maximus. These muscles were selected to represent the principal lower-limb muscle groups involved in STS transfer. The vastus lateralis and vastus medialis were included as primary knee extensors during the rising phase; the biceps femoris was included to represent posterior thigh activity and hip/knee control during transition; the gluteus maximus was included as a primary hip extensor during rising; and the gluteus medius was included because of its role in frontal-plane pelvic and hip stabilization during transitional movement. The repeated STS task, bilateral EMG electrode placement, and data-processing workflow are illustrated in Figure 1. Participants wore tight-fitting clothing during data collection to minimize motion artifacts. Before electrode placement, the skin was prepared using alcohol wipes to reduce impedance. Muscle locations were identified through palpation, and electrode locations were identified according to standardized surface EMG placement recommendations for the respective muscles, with palpation used to locate the muscle belly and avoid tendon regions; placement was further verified during MVC testing by visual inspection of signal amplitude and quality [22].

### 2.3. Data Processing

Raw EMG signals were processed using MATLAB R2024b (MathWorks Inc., Natick, MA, USA). Signals were band-pass-filtered using a fourth-order Butterworth filter (20–450 Hz) to attenuate low-frequency motion artifacts and high-frequency noise, then full-wave-rectified and passed through a low-pass fourth-order Butterworth filter with a cutoff frequency of 20 Hz to generate linear EMG envelopes. Participants performed three maximum voluntary contraction (MVC) trials for each muscle group to establish reference activation levels for normalization. The peak activation value across the three trials was used as the MVC reference for each muscle. Processed EMG signals were normalized to MVC peak activation to express STS-related muscle activation relative to each participant’s own maximum voluntary activation capacity. MVC normalization was selected to reduce inter-subject variability arising from electrode placement, skin impedance, and raw signal scaling and to allow comparison of relative neuromuscular demand across participants and groups. Alternative normalization approaches, such as normalization to task-specific peak activation or sub-maximal reference contractions, were not formally compared in the present study.

Repeated STS transfers were segmented into sit-to-stand and stand-to-sit phases based on the observed task sequence and corresponding EMG recordings. The sit-to-stand phase was defined as the movement from seated to upright standing, and the stand-to-sit phase as the movement from upright standing to seated posture. Marker-derived kinematic variables were not used for phase segmentation in the present analysis. Normalized EMG amplitudes were initially quantified bilaterally for all five muscle groups during each phase. For each participant, left- and right-side values were averaged for each muscle and phase to obtain a single participant-level value. Bilateral recordings were therefore not treated as independent observations. Mean normalized EMG amplitudes were calculated across the five repetitions for each participant and used in all subsequent analyses. The present analysis focused on phase-specific normalized EMG amplitude as a measure of gross neuromuscular activation during repeated STS transfers. Temporal activation features, onset/offset timing, frequency-domain characteristics, co-activation indices, and repetition-to-repetition variability were not analyzed in this study.

### 2.4. Statistical Analysis

Statistical analyses were performed using IBM SPSS Statistics for Windows Version 23.0 (IBM Corp., Armonk, NY, USA). Descriptive statistics are presented as mean ± standard deviation. Independent-sample *t*-tests were used to compare normalized EMG amplitudes between the DPN and control groups during the STS and stand-to-sit phases. Paired-sample *t*-tests were used to assess phase-dependent activation differences within each group. Because of the modest sample size and exploratory nature of the study, the results of parametric tests were interpreted with caution. Formal assumption testing for normality and homogeneity of variance was not included in the original analysis workflow; therefore, statistical inference was based on the combined interpretation of *p*-values, effect sizes, and 95% confidence intervals rather than *p*-values alone. No formal multiple-comparison correction was applied; accordingly, muscle-wise *p*-values were treated as exploratory and interpreted considering the number of comparisons performed. The large variability observed for biceps femoris activation during the stand-to-sit phase in the DPN group may reflect true inter-individual variability, signal variability, or possibly influential observations. Because no predefined outlier-exclusion or sensitivity-analysis procedure was applied, this finding should be interpreted cautiously. Effect sizes were calculated as Cohen’s d for all comparisons to characterize effect magnitude independent of sample size. For between-group comparisons, 95% confidence intervals were calculated for mean differences to characterize uncertainty around the estimated group differences. Statistical significance was set at *p* < 0.05, with emphasis placed on effect magnitude, 95% confidence intervals, and consistency of findings rather than *p*-values alone.

## 3. Results

### 3.1. Participant Demographics

Group demographics were earlier presented in Table 1. No significant between-group differences were observed for age, height, weight, or BMI (all *p* > 0.050), confirming the adequacy of group matching.

### 3.2. Between-Group Comparisons of Normalized EMG Amplitude

Normalized EMG amplitudes during repeated STS transfers were compared between the DPN and control groups for both phases. No statistically significant between-group differences were observed for any muscle during either phase (all *p* > 0.050). Results are presented in Table 2 and Table 3. These non-significant findings should be interpreted as absence of statistically detectable differences in this sample rather than evidence of equivalence between groups.

**Figure 2:**
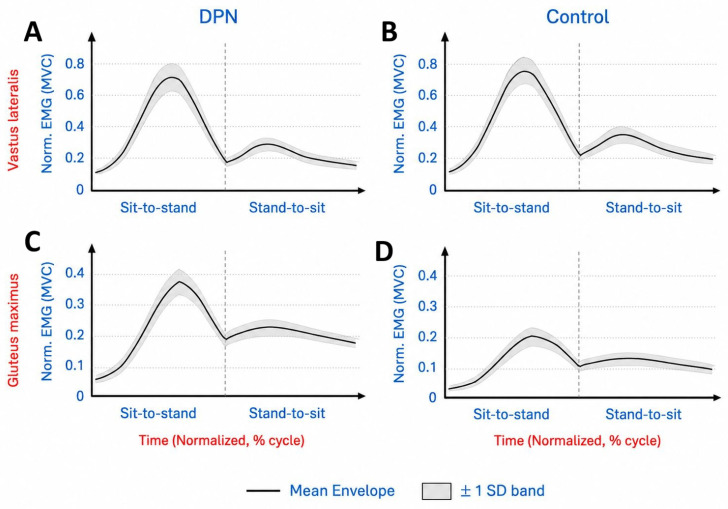
Representative normalized electromyographic (EMG) envelopes during repeated STS and stand-to-sit phases for the vastus lateralis and gluteus maximus in patients with DPN and healthy controls. (**A**) Vastus lateralis—DPN. (**B**) Vastus lateralis—Control. (**C**) Gluteus maximus—DPN. (**D**) Gluteus maximus—Control. Solid lines represent group mean normalized EMG amplitude (fraction of maximum voluntary contraction, MVC); shaded bands represent ±1 standard deviation. The vertical dashed line denotes the transition between the sit-to-stand and stand-to-sit phases. Waveforms are based on group mean amplitudes derived from the normalized EMG data across five repeated trials per participant.

Group comparisons of normalized EMG amplitudes during the STS and stand-to-sit phases are illustrated in Figure 2. During the STS phase, vastus lateralis activation was 0.337 ± 0.259 in the DPN group and 0.368 ± 0.277 in controls (*p* = 0.754; d = 0.11). Vastus medialis activation was 0.327 ± 0.171 and 0.382 ± 0.207, respectively (*p* = 0.441; d = 0.29). Biceps femoris activation was 0.135 ± 0.104 in the DPN group and 0.188 ± 0.170 in controls (*p* = 0.315; d = 0.37). Gluteus maximus activation was 0.241 ± 0.208 in the DPN group and 0.143 ± 0.125 in controls (*p* = 0.130; d = 0.54), representing the largest between-group effect size observed and the only comparison approaching a medium effect. Gluteus medius activation was comparable between groups (DPN: 0.120 ± 0.059; controls: 0.113 ± 0.068; *p* = 0.756; d = 0.11).

During the stand-to-sit phase, no significant between-group differences were identified for any muscle. Vastus lateralis activation was 0.209 ± 0.123 in the DPN group and 0.279 ± 0.270 in controls (*p* = 0.375; d = 0.33). Vastus medialis activation was 0.316 ± 0.282 and 0.343 ± 0.234, respectively (*p* = 0.779; d = 0.10). Biceps femoris activation during the stand-to-sit phase showed marked variability in the DPN group (0.278 ± 0.731) compared with controls (0.257 ± 0.263; *p* = 0.917; d = 0.04). No outlier exclusion or sensitivity analysis was performed for this comparison because outlier-removal criteria were not predefined. Therefore, this result should be interpreted as indicating substantial inter-individual variability rather than a stable between-group difference. Gluteus maximus activation was 0.130 ± 0.126 in the DPN group and 0.105 ± 0.095 in controls (*p* = 0.543; d = 0.22), and gluteus medius activation was 0.117 ± 0.135 and 0.121 ± 0.164, respectively (*p* = 0.938; d = 0.03).

Effect-size estimates for most between-group comparisons were small, with confidence intervals indicating considerable uncertainty around the estimated differences. The largest between-group effect was observed for gluteus maximus activation during the STS phase (d = 0.54), suggesting a possible moderate difference despite the absence of statistical significance. Given the modest sample size and the uncertainty around the estimated effects, smaller but clinically meaningful between-group differences could not be confirmed or ruled out.

### 3.3. Within-Group Phase-Dependent Comparisons

**Figure 3:**
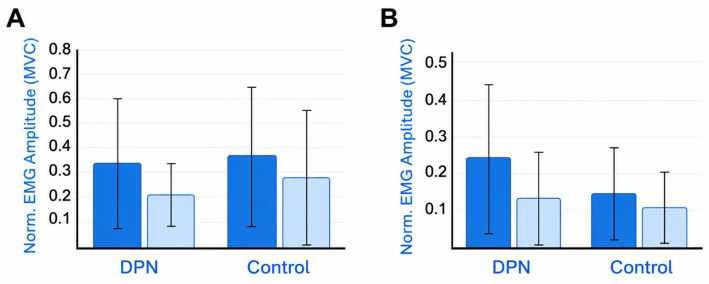
Normalized electromyographic (EMG) amplitudes for the (**A**) vastus lateralis and (**B**) gluteus maximus during STS (Dark Blue) and stand-to-sit (Light Blue) phases in patients with DPN and healthy controls. Values are presented as mean normalized amplitude (fraction of maximum voluntary contraction, MVC). Error bars represent ±1 standard deviation. Significance markers denote within-group phase differences (paired-samples *t*-test): *p* < 0.050; *p* < 0.010; *p* < 0.001. No significant between-group differences were observed for either muscle during either phase (all *p* > 0.050).

Both groups demonstrated significantly greater muscle activation during the STS phase compared with the stand-to-sit phase for the vastus lateralis and gluteus maximus. Results are presented in Table 4. Phase-dependent activation differences for the vastus lateralis and gluteus maximus are highlighted in Figure 3.

In the DPN group, vastus lateralis activation increased significantly from 0.209 ± 0.123 during stand-to-sit to 0.337 ± 0.259 during STS (*p* = 0.009; d = 0.63). Gluteus maximus activation also increased significantly during the rising phase, from 0.130 ± 0.126 to 0.241 ± 0.208 (*p* = 0.001; d = 0.64). No significant phase-dependent differences were observed for the vastus medialis, biceps femoris, or gluteus medius in the DPN group (all *p* > 0.050). In the control group, vastus lateralis activation increased significantly from 0.279 ± 0.270 during stand-to-sit to 0.368 ± 0.277 during STS (*p* = 0.007; d = 0.33). Gluteus maximus activation increased from 0.105 ± 0.095 to 0.143 ± 0.125 (*p* = 0.013; d = 0.35). No significant phase-dependent differences were observed for the vastus medialis, biceps femoris, or gluteus medius in the control group (all *p* > 0.050). The effect sizes for phase-dependent vastus lateralis and gluteus maximus differences were larger in the DPN group (d = 0.63–0.64) than in the control group (d = 0.33–0.35), suggesting greater relative neuromuscular demand during the rising phase in patients with DPN despite similar absolute activation amplitudes between groups.

## 4. Discussion

This study examined lower-limb muscle activation patterns during repeated STS transfers in ambulatory patients with DPN compared with age- and gender-matched healthy controls, with the aim of determining whether MVC-normalized sEMG amplitude is sensitive to neuropathic neuromuscular impairment during this transitional task. No statistically significant between-group differences in normalized EMG amplitudes were observed for any muscle during either phase. Both groups demonstrated significantly greater vastus lateralis and gluteus maximus activation during the STS phase compared with stand-to-sit. These findings are discussed below in the context of DPN-related neuromuscular impairment, compensatory motor strategies, and the clinical utility of amplitude-based sEMG metrics during transitional tasks.

The present analysis extends our prior work in the same cohort by shifting the functional context from gait and postural control to repeated STS transfers and focusing specifically on phase-dependent lower-limb EMG amplitude. This distinction is important because STS is a transitional task requiring coordinated rising and lowering movements, rather than steady-state locomotion. Therefore, the added value of the present study lies in testing whether amplitude-based sEMG during a clinically common transitional task provides additional information about neuromuscular function in ambulatory patients with DPN.

### 4.1. Preserved Amplitude and the Sensitivity of Normalized EMG in Ambulatory DPN

The absence of statistically significant between-group differences in normalized EMG amplitude does not imply equivalence between groups or intact neuromuscular function in DPN. Instead, these findings indicate that statistically detectable amplitude differences were not observed in this modestly sized ambulatory cohort, and the results should be interpreted in the context of effect sizes, confidence intervals, and the exploratory design of the study. As reviewed by Allen et al. [7], motor unit loss in DPN follows a length-dependent progression and typically occurs later in the disease process, after the onset of sensory deficits. In ambulatory patients with preserved functional mobility, surviving motor units undergo compensatory reinnervation and may upregulate discharge rates to maintain gross force output, resulting in comparable normalized EMG amplitudes despite underlying neuropathic impairment. This mechanistic framework predicts that amplitude metrics would have limited sensitivity for detecting early-to-moderate DPN-related neuromuscular changes, a prediction consistent with the present findings.

This interpretation is supported by the pattern of effect sizes and the uncertainty reflected in the 95% confidence intervals. The majority of between-group comparisons yielded small effect sizes, suggesting that true neuromuscular differences, if present, are likely small in magnitude in this ambulatory cohort and would require substantially larger samples to confirm. These findings are consistent with our previous work in the same cohort, in which dynamic postural stability during walking was relatively preserved despite impairments in static balance and gait velocity [13]; collectively, they support the possibility that ambulatory patients with DPN adopt compensatory movement strategies that permit maintenance of functional performance metrics despite underlying sensorimotor degradation.

The present findings also have direct implications for clinical sEMG assessment. Normalized amplitude during repeated STS transfers does not appear to be a sensitive discriminator between ambulatory DPN patients and healthy controls of similar age and functional status. Clinicians and researchers using sEMG to characterize neuromuscular function in DPN during transitional tasks should consider complementing amplitude analysis with metrics more sensitive to the sensory-dominant early stages of neuropathic impairment, including activation timing, muscle co-activation ratios, intermuscular coordination, and motor output variability across repeated trials [11,12]. Recent DPN studies further demonstrate that manipulation of plantar sensory input can influence standing balance, gait quality, and ankle-foot muscle activity, reinforcing the value of combining EMG amplitude with task-specific sensory and biomechanical measures [23,24]. Evidence from other clinical populations similarly supports the value of phase-specific EMG assessment. In individuals with chronic low back pain, dynamic neuromuscular stabilization training was associated with selective changes in lower-limb activation across distinct gait phases, including the vastus medialis and biceps femoris [25].

### 4.2. Gluteus Maximus Activation as a Hypothesis-Generating Finding

The most notable hypothesis-generating finding was the greater gluteus maximus activation observed in the DPN group during the STS phase (0.241 ± 0.208 vs. 0.143 ± 0.125; *p* = 0.130; d = 0.54). This comparison did not reach statistical significance and should not be interpreted as evidence of a confirmed between-group difference. However, because it represented the largest effect size observed in the study, it may be clinically relevant and warrants further investigation in adequately powered studies.

Biomechanically, one possible explanation is that greater hip extensor recruitment during STS rising may reflect a proximal motor-control strategy in which patients with DPN rely more heavily on hip musculature during the transition from sitting to standing. DPN-related sensory impairment is length-dependent and affects distal nerve fibers first, meaning that proximal hip musculature may retain more reliable sensorimotor control than distal quadriceps or ankle stabilizers [2,7]. A proximal compensation strategy during STS, characterized by greater gluteus maximus engagement, would therefore be mechanistically coherent with the known sensory distribution of DPN. Similar proximal compensation patterns have been proposed in the DPN gait literature, where patients demonstrate altered hip and trunk strategies to compensate for impaired ankle and foot proprioception [2,10]. Although derived from a different population and functional task, findings from single-leg landing indicate that proximal hip-flexor restriction can alter gluteus maximus and bicep femoris activation across feed-forward and feedback periods, illustrating how proximal mechanical constraints may influence lower-extremity recruitment through the kinetic chain [26]. However, the present data cannot determine whether this pattern reflects true compensation, altered movement kinematics, inter-individual variability, or another mechanism. This interpretation should therefore be treated as a hypothesis for future work using larger samples and concurrent EMG-kinematic analysis.

### 4.3. Phase-Dependent Neuromuscular Demand

Both groups showed significantly greater vastus lateralis and gluteus maximus activation during STS than stand-to-sit, consistent with the elevated knee and hip extensor demands of rising against gravity [15] and confirming that this phase-dependent pattern is preserved in ambulatory DPN. Notably, within-group effect sizes were larger in the DPN group (vastus lateralis: d = 0.63; gluteus maximus: d = 0.64) than in the control group (d = 0.33; d = 0.35), suggesting greater relative neuromuscular effort during rising despite comparable absolute amplitudes, a pattern possibly reflecting impaired anticipatory postural adjustments or less economical center-of-mass transfer mechanics secondary to proprioceptive deficits, though kinematic confirmation is required. No phase-dependent differences were observed for the vastus medialis, biceps femoris, or gluteus medius in either group, indicating that phase-specific neuromuscular demand during STS is concentrated in the primary sagittal-plane extensors.

From a clinical perspective, these findings support the continued use of repeated STS tasks as practical assessments of transitional mobility in patients with DPN, but also suggest that MVC-normalized EMG amplitude should not be used as a standalone marker of neuropathic neuromuscular impairment. Furthermore, clinical assessment protocols may be strengthened by combining amplitude-based EMG with activation timing, co-contraction, bilateral symmetry, repetition-to-repetition variability, and kinematic measures of trunk and lower-limb movement. For rehabilitation research, repeated STS tasks may provide a useful framework for examining rising and lowering control, knee and hip extensor demand, and potential compensatory strategies, although the present findings should be treated as exploratory rather than as direct evidence for a specific rehabilitation intervention. Recent randomized trials support the responsiveness of balance and mobility outcomes in DPN to targeted rehabilitation. Computer-based balance training and foot-ankle therapeutic exercise have improved postural stability, fall-risk measures, or gait speed [27,28], while sensorimotor, multisystem, proprioceptive, and somatosensory-focused programs have also improved gait or balance-related outcomes [29,30,31,32].

### 4.4. Limitations

Several limitations should be considered when interpreting these findings. First, the study participants comprised ambulatory, functionally independent DPN patients, limiting generalizability to more severe or sedentary populations. Potential sources of bias include the small convenience sample, recruitment of ambulatory and functionally independent DPN participants, lack of neuropathy-severity stratification, and unmeasured factors such as physical activity level and treatment status. These factors may have influenced the observed EMG amplitudes and may limit generalizability to patients with more severe neuropathy or lower functional capacity. The DPN participants were analyzed as a single group, and the study did not include a non-neuropathic diabetic comparison group. In addition, the sample size was insufficient to stratify participants by neuropathy severity. Therefore, the present findings may not capture severity-dependent neuromuscular differences across the DPN spectrum. Future studies should include non-neuropathic diabetic participants and mild, moderate, and severe DPN subgroups to determine whether phase-specific STS activation patterns vary with neuropathy progression.

The modest sample size limited the ability to detect small-to-medium between-group effects; therefore, the null between-group findings should be interpreted as inconclusive with respect to equivalence rather than as evidence that DPN and control participants had truly similar EMG activation patterns. Side-to-side differences in EMG activation were not analyzed because bilateral recordings averaged for the primary group-level analysis. Therefore, the present results do not address potential limb asymmetry in patients with DPN, which may be clinically relevant and should be examined in future studies using side-specific analysis.

Chair height was fixed regardless of participant stature, and knee angle at seat-off was not controlled or included as a covariate. This may have introduced variability in hip and knee joint angles and could have influenced quadriceps and gluteal activation amplitudes, particularly during the rising phase.

Marker-derived kinematic variables were not used to define phase-transition events in the present EMG analysis. Therefore, precise biomechanical events such as seat-off, peak hip/knee extension, movement initiation, and return-to-seat could not be examined. This limits the ability to relate EMG amplitude changes directly to joint mechanics, trunk motion, or center-of-mass displacement during STS and stand-to-sit transitions. Future studies should integrate synchronized EMG and kinematic event detection to improve phase-specific interpretation. Because the present analysis was limited to normalized EMG amplitude, it might not have detected DPN-related alterations in activation timing, motor coordination, co-contraction patterns, motor-output variability, or frequency-domain characteristics. Future studies should incorporate temporal, frequency-domain, and intermuscular coordination metrics to provide a more complete characterization of neuromuscular control during STS transfers.

MVC normalization may also obscure differences in absolute muscle activation or force-generating capacity, particularly in clinical populations such as DPN where maximal voluntary activation may itself be impaired. Therefore, the normalized EMG values in this study should be interpreted as relative activation amplitudes rather than direct indicators of absolute muscle force. Self-selected movement pace may have introduced inter-individual variability in neuromuscular demand. Finally, accompanying kinematic data were not analyzed for this study; integration of joint angle and center-of-mass trajectories with EMG in future work would substantially strengthen phase-specific interpretation.

## 5. Conclusions

In this exploratory study, no statistically significant between-group differences in MVC-normalized lower-limb EMG amplitude were observed between ambulatory patients with DPN and healthy controls during repeated STS transfers. However, these null findings should not be interpreted as evidence of equivalence or normal neuromuscular function in DPN. Given the modest sample size and the uncertainty around the estimated effects, smaller but clinically meaningful between-group differences could not be confirmed or ruled out. The largest between-group effect was observed for gluteus maximus activation during the rising phase (Cohen’s d = 0.54), but this finding was not statistically significant and should be interpreted as hypothesis-generating. It may indicate greater reliance on hip-extensor activity during STS in some patients with DPN, but confirmation requires larger studies incorporating concurrent kinematic analysis. This is consistent with the known pattern of DPN, in which nerve damage progresses from distal to proximal, leaving proximal muscles better preserved and potentially overloaded in compensatory movements. These findings warrant follow-up in larger studies that also integrate movement kinematics.

Both groups activated the vastus lateralis and gluteus maximus significantly more during rising than during lowering, confirming that standing up is the more demanding phase of the task. Notably, this phase-related increase was larger in the DPN group, suggesting that patients with DPN may experience greater relative neuromuscular demand in rising from a seat despite displaying comparable normalized EMG magnitudes.

From a clinical standpoint, this study shows that measuring EMG amplitude alone during STS transfers is not sensitive enough to detect neuromuscular impairment in ambulatory patients with DPN. Clinicians and researchers should consider adding measures of muscle activation timing, co-activation between muscle pairs, bilateral symmetry, and trial-to-trial consistency to their assessment toolkit. These metrics are better suited to capturing the early neuromuscular changes associated with DPN. Repeated STS testing remains a practical and valuable clinical tool, and its true potential for detecting motor control deficits in DPN will be best realized through more comprehensive neuromuscular assessment in larger future studies.

## Figures and Tables

**Table 1:** Demographics of study participants.

Characteristic	DPN Group (n = 15)	Control Group (n = 15)
Gender (M/F)	11/4	11/4
Age (years)	61.50 ± 7.97	59.13 ± 7.85
Height (m)	1.70 ± 0.08	1.75 ± 0.09
Weight (kg)	83.66 ± 12.21	81.54 ± 20.42
BMI (kg/m^2^)	28.92 ± 4.46	26.31 ± 4.66
Diabetes type	Type II	-
HbA1c (%)	8.20 ± 1.40	-
Diabetes duration (years)	14.33 ± 4.98	-

BMI: body mass index; HbA1c: glycated hemoglobin; DPN: diabetic peripheral neuropathy. Data are presented as mean ± standard deviation unless otherwise stated.

**Table 2:** Normalized EMG amplitudes during the sit-to-stand phase.

Muscle	DPN (Mean ± SD)	Control (Mean ± SD)	Mean Difference (95% CI)	*p*-Value	Cohen’s d (95% CI)
Vastus lateralis	0.337 ± 0.259	0.368 ± 0.277	−0.031 (−0.232 to 0.170)	0.754	−0.11 (−0.83 to 0.61)
Vastus medialis	0.327 ± 0.171	0.382 ± 0.207	−0.055 (−0.197 to 0.087)	0.441	−0.29 (−1.01 to 0.43)
Biceps femoris	0.135 ± 0.104	0.188 ± 0.170	−0.053 (−0.158 to 0.052)	0.315	−0.37 (−1.09 to 0.35)
Gluteus maximus	0.241 ± 0.208	0.143 ± 0.125	0.098 (−0.030 to 0.226)	0.130	0.54 (−0.19 to 1.27)
Gluteus medius	0.120 ± 0.059	0.113 ± 0.068	0.007 (−0.041 to 0.055)	0.756	0.11 (−0.61 to 0.83)

Normalized EMG amplitudes are presented as mean ± standard deviation. No significant between-group differences were observed (all *p* > 0.050). Cohen’s d is reported for all comparisons to characterize effect magnitude independent of sample size. Cohen’s d values are reported as signed effects calculated as DPN–Control; 95% confidence intervals are provided to indicate uncertainty around the estimated effect sizes.

**Table 3:** Normalized EMG amplitudes during the stand-to-sit phase.

Muscle	DPN (Mean ± SD)	Control (Mean ± SD)	Mean Difference (95% CI)	*p*-Value	Cohen’s d (95% CI)
Vastus lateralis	0.209 ± 0.123	0.279 ± 0.270	−0.070 (−0.227 to 0.087)	0.375	−0.33 (−1.05 to 0.39)
Vastus medialis	0.316 ± 0.282	0.343 ± 0.234	−0.027 (−0.221 to 0.167)	0.779	−0.10 (−0.82 to 0.62)
Biceps femoris	0.278 ± 0.731 ^†^	0.257 ± 0.263	0.021 (−0.390 to 0.432)	0.917	0.04 (−0.68 to 0.76)
Gluteus maximus	0.130 ± 0.126	0.105 ± 0.095	0.025 (−0.059 to 0.109)	0.543	0.22 (−0.50 to 0.94)
Gluteus medius	0.117 ± 0.135	0.121 ± 0.164	−0.004 (−0.116 to 0.108)	0.938	−0.03 (−0.75 to 0.69)

Normalized EMG amplitudes are presented as mean ± standard deviation. No significant between-group differences were observed (all *p* > 0.050). Cohen’s d is reported for all comparisons. Cohen’s d values are reported as signed effects calculated as DPN – Control; 95% confidence intervals are provided to indicate uncertainty around the estimated effect sizes. ^†^ Denotes high inter-individual variability in the DPN group for this muscle during the stand-to-sit phase.

**Table 4:** Within-group phase-dependent comparison of normalized EMG amplitudes.

Muscle	Sit-to-Stand (Mean ± SD)	Stand-to-Sit (Mean ± SD)	*p*-Value	Cohen’s d
**DPN Group**				
Vastus lateralis	0.337 ± 0.259	0.209 ± 0.123	0.009 *	0.63
Vastus medialis	0.327 ± 0.171	0.316 ± 0.282	>0.050	0.05
Biceps femoris	0.135 ± 0.104	0.278 ± 0.731	>0.050	0.27
Gluteus maximus	0.241 ± 0.208	0.130 ± 0.126	0.001 *	0.64
Gluteus medius	0.120 ± 0.059	0.117 ± 0.135	>0.050	0.03
**Control Group**				
Vastus lateralis	0.368 ± 0.277	0.279 ± 0.270	0.007 *	0.33
Vastus medialis	0.382 ± 0.207	0.343 ± 0.234	>0.050	0.18
Biceps femoris	0.188 ± 0.170	0.257 ± 0.263	>0.050	0.32
Gluteus maximus	0.143 ± 0.125	0.105 ± 0.095	0.013 *	0.35
Gluteus medius	0.113 ± 0.068	0.121 ± 0.164	>0.050	0.07

Data are presented as mean ± standard deviation. Cohen’s d was calculated using pooled standard deviation for paired comparisons. * Statistically significant at *p* < 0.050. Activation was greater during STA than during stand-to-sit.

## Data Availability

The data presented in this study are available upon reasonable request from the corresponding author.

## Abbreviations

The following abbreviations are used in this manuscript:

BMI	Body Mass Index
COVID	Coronavirus Disease 2019
DPN	Diabetic Peripheral Neuropathy
EMG	Electromyography
HbA1c	Hemoglobin A1c
IBM	International Business Machines Corporation
SPSS	Statistical Package for the Social Sciences
IRB	Institutional Review Board
MVC	Maximum Voluntary Contraction
sEMG	Surface Electromyography
STS	Sit-to-Stand
UALR	University of Arkansas at Little Rock
UAMS	University of Arkansas for Medical Sciences
